# Advancements in chimeric antigen receptor-expressing T-cell therapy for glioblastoma multiforme: Literature review and future directions

**DOI:** 10.1093/noajnl/vdae025

**Published:** 2024-02-20

**Authors:** Michael Goutnik, Alexandria Iakovidis, Megan E H Still, Rachel S F Moor, Kaitlyn Melnick, Sandra Yan, Muhammad Abbas, Jianping Huang, Ashley P Ghiaseddin

**Affiliations:** Department of Neurosurgery, College of Medicine, University of Florida, Gainesville, Florida, USA; Department of Neurosurgery, College of Medicine, University of Florida, Gainesville, Florida, USA; Department of Neurosurgery, College of Medicine, University of Florida, Gainesville, Florida, USA; Department of Neurosurgery, College of Medicine, University of Florida, Gainesville, Florida, USA; Department of Neurosurgery, College of Medicine, University of Florida, Gainesville, Florida, USA; Department of Neurosurgery, College of Medicine, University of Florida, Gainesville, Florida, USA; Department of Neurosurgery, College of Medicine, University of Florida, Gainesville, Florida, USA; Department of Neurosurgery, College of Medicine, University of Florida, Gainesville, Florida, USA; Department of Neurosurgery, College of Medicine, University of Florida, Gainesville, Florida, USA

**Keywords:** antigen escape, CAR-T cells, glioblastoma multiforme, immunotherapy, tumor microenvironment

## Abstract

Glioblastoma multiforme (GBM) is an aggressive cancer that has been difficult to treat and often requires multimodal therapy consisting of surgery, radiotherapy, and chemotherapy. Chimeric antigen receptor-expressing (CAR-T) cells have been efficacious in treating hematological malignancies, resulting in several FDA-approved therapies. CAR-T cells have been more recently studied for the treatment of GBM, with some promising preclinical and clinical results. The purpose of this literature review is to highlight the commonly targeted antigens, results of clinical trials, novel modifications, and potential solutions for challenges that exist for CAR-T cells to become more widely implemented and effective in eradicating GBM.

**Key Points**

- Recent preclinical and clinical studies have yielded promising results for chimeric antigen receptor-expressing-T treatment of glioblastoma multiforme.
- Future studies will likely include combination treatment with other immunotherapy approaches.

Glioblastoma multiforme (GBM) is an aggressive central nervous system malignancy with a dismal median overall survival of 15 months and <5% 5-year survival.^1,2^ In the United States, the incidence is 3.19/100 000, with the highest incidence in the 8th decade of life and 1.6 times higher incidence in males.^2,3^ Clinical presentation varies based on the location of the tumor.^4^ The Stupp protocol, which consists of surgical resection and radiotherapy with concomitant and adjuvant temozolomide, is the current standard of care for newly diagnosed GBM.^5^ Outside of the addition of tumor-treating fields, the standard of care has remained unchanged since the Stupp protocol gained acceptance nearly 20 years ago.^6^ A variety of immunotherapies are also emerging as promising treatment modalities for various malignancies but have been more challenging to implement in GBMs, which are often resistant to immunotherapeutic treatments.^3,7^ One promising type of immunotherapy involves chimeric antigen receptors (CARs), which are genetically engineered receptors that redirect lymphocytes to recognize and bind to desired antigens.^8^ They consist of an extracellular antigen binding region, an extracellular hinge region, a transmembrane domain, and an intracellular signaling domain.^9^ The original generation of CARs had an extracellular antigen-recognizing domain coupled with a single intracellular domain (CD3z) for signal transduction.^10^ In an attempt to improve inadequate persistence of the cells in solid tumors, second-generation constructs featured a costimulatory signaling domain (typically CD28 or 4-1BB) in addition to the CD3z intracellular domain.^10^ Further attempts to increase intratumoral persistence and efficacy led to third-generation constructs, which feature 2 costimulatory domains. Finally, among other strategies, newer generations of CARs aim to exploit properties of tumor microenvironments, such as reliance on immunosuppressive chemokine signaling.^10,11^ CARs are particularly useful because they do not require antigen processing for presentation by human leukocyte antigens.^12^ CAR-T cells are created via apheresis of patients’ T-cells, followed by ex vivo genetic modification. This is followed by expansion of the modified T-cells in culture prior to patient infusion (Figure 1).^13^

**Figure 1.**
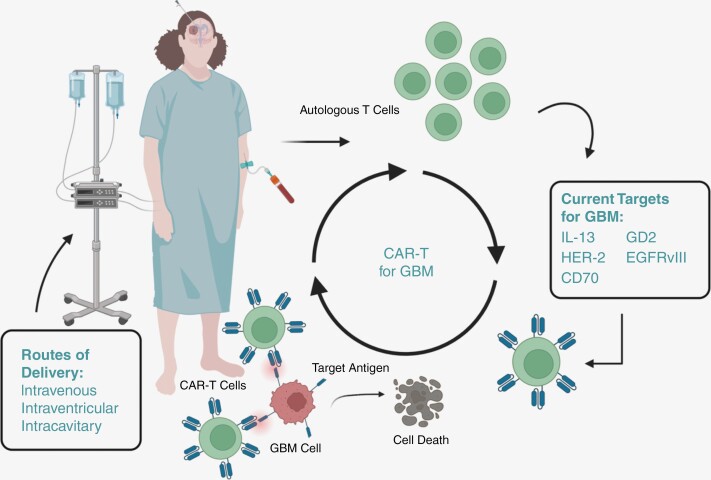
Visual synopsis of current chimeric antigen receptor-expressing-T strategies for the treatment of glioblastoma.

There have been 6 FDA-approved CAR-T cell therapies. The 6 therapies and trials are summarized in Table 1. Four of these—Tisagenlecleucel, Axicabtagene ciloleucel, Brexucabtagene autoleucel, and Lisocabtagene maraleucel—target CD-19. The other 2—Idecabtagene vicleucel and Ciltacabtagene autoleucel—target B-cell maturation antigen (BCMA), a protein expressed by both malignant and normal plasma cells.^14^ While CAR-T cell therapy has shown promising results in hematologic malignancies, it has not been without adverse effects.^15^ Cytokine release syndrome (CRS) is the most common of the adverse effects experienced among patients.^16^ CRS ranges from flu-like symptoms, such as fever, hypotension, myalgias, and arthralgias, to multi-organ dysfunction. Symptoms can develop within the first week following CAR-T cell administration but can occur up to several weeks after.^17^

**Table 1.** FDA-approved CAR-T therapies.

CD-19 CARS	Year approved	Disease	Results	Cytokine release syndrome prevalence
Axicabtagene ciloleucel^45^ (NCT02348216)	2017	Relapsed or refractory large B-cell lymphoma	51% of patients (*n* = 101) had complete remission	93%
Tisagenlecleucel^47^ (NCT02435849)	2017	Relapsed or refractory B-cell acute lymphoblastic leukemia (B-ALL)	81% of patients (*n* = 75) had remission	79%
Brexucabtagene autoleucel^48^ (NCT02614066)	2021	B-ALL	52% of patients (*n* = 54) had complete remission	89%
Lisocabtagene maraleucel^49^ (NCT02631044)	2022	Relapsed or refractory large B-cell lymphoma	53% of patients (*n* = 269) had complete response	42%
BCMA CARS				
Idecabtagene vicleucel^50^ (NCT03361748)	2021	Relapsed/refractory multiple myeloma (RRMM)	28% of patients (*n* = 100) had a complete response	84%
Ciltacabtagene autoleucel^51^ (NCT03548207)	2022	RRMM	67% of patients (*n* = 97%) achieved complete response	95%

CARs targeting GBM-specific antigens were first described in 2004.^18^ Years of studying the application of CAR-T cells to treat GBM have revealed many challenges, including antigen specificity, hostile tumor microenvironment, and limited penetration.^19^ However, iterative generations of CAR-T cells with new targets, constructs, and therapeutic combinations continue to be developed for GBM, with the goal of improving outcomes in the lethal disease. The purpose of this review is to highlight the application of CAR-T therapy to GBM and the obstacles that need to be overcome in the current landscape.

## Ongoing Research Efforts

### Preclinical Studies

Several preclinical studies have provided the basis for CAR-T clinical trials in gliomas, with a few important implicated receptors. For example, it has been previously shown that interleukin-13 (IL-13) receptors—particularly IL-13 receptor alpha2 (IL-13Rα2)—are highly expressed in, and restricted to, gliomas and not normal brain parenchyma or surrounding cells.^18,20,21^ The receptor is also associated with mesenchymal features and poor survival, which makes it an important therapeutic target.^22^ In 2004, Kahlon et al. created the first GBM CAR construct, a mutant IL-13-zetakine CTL (cytotoxic T lymphocyte) that specifically targeted IL-13Rα2 in GBM tumor cells.^18^ This mutant selectively targeted tumor cells expressing IL-13Rα2 without affecting normal cells expressing IL-13Rα1, which reduced unwanted side effects. The intracranial administration of IL-13Rα2-specific CAR-T cells into orthotopic GBM mice successfully resulted in tumor regression. Following this study, in 2012, Brown et al. showed that IL-13Rα2 was expressed in both glioma stem cell (GSC) and more differentiated cell populations with equal frequency.^23^ The rationale for targeting GSCs is their likely role in glioma initiation and tumor recurrence.^24^ Brown et al. showed that IL-13-zetakine CTLs administered intracranially were able to recognize and destroy GBM cells, including GSCs, in vitro and in orthotopic mice, with resultant improved survival.^23^ To address inadequate T cell persistence, Brown et al. added a costimulatory signaling domain 4-1BB (CD137), and these second-generation CAR-T cells improved survival and enhanced antitumor activity in orthotopic mice.^25^ In addition, low-dose (0.2 mg/kg) dexamethasone, which is commonly given to patients with GBM to treat edema, did not significantly impact the efficacy of the CAR-T cells. These results were encouraging and provided a rationale for clinical trials targeting the mutant IL-13 receptor.

Another commonly cited receptor is the epidermal growth factor receptor (EGFR), with approximately 30% of gliomas expressing the mutant EGFR—EGFRvIII.^26^ There have been numerous attempts to target the aberrant receptor using monoclonal antibodies and small-molecule inhibitors.^27^ In 2009, Bullain et al. developed genetically engineered T cells expressing a chimeric T cell receptor that targeted EGFRvIII.^28^ Their construct included a tumor antigen binding domain, transmembrane linker, and a cytoplasmic T cell receptor signaling domain (CD3 zeta). The engineered cells showed in vitro efficacy against mutant EGFR and not wild-type EGFR-expressing glioma cell lines. However, in vivo activity in a murine model was not as efficacious, leading to the hypothesis that incorporating costimulatory signaling domains (such as CD28) in the construct might improve the immune response. In 2012, a similar study focused on targeting glioma stem cells (GSCs), which were hypothesized to be a more accurate model for studying responses in GBM, compared to established cell lines.^29^ The authors genetically modified T cells (third-generation construct, which included additional CD28 and 4-1BB signaling domains that have been shown to improve CAR-T survival, cytokine release, and tumor eradication^30^) to target GSCs expressing EGFRvIII. These engineered T cells were able to produce cytokines and destroy EGFRvIII-expressing GSC lines without causing any adverse effects on healthy cells. A subsequent study built from these in vitro studies in immunodeficient murine model studies by employing immunocompetent mice to create a murine EGFRvIII-CAR.^31^ The authors found that lymphodepletive conditioning via irradiation was necessary for EGFRvIII-CAR efficacy. In this study, mice receiving EGFRvIII-CAR-T cells were cured and developed resistance to forming new EGFRvIII-expressing tumors when additional EGFRvIII-positive tumor was subcutaneously injected, which suggested durable anti-EGFRvIII activity. Finally, peripheral EGFRvIII-CAR-T cell levels persisted for 5 weeks throughout the experiment. In 2014, another study attempted to recapitulate the immune environment and invasive characteristics of GBM by using a xenograft with a patient-derived primary GBM cell line expressing EGFRvIII (D-270 MG).^32^ The authors then created EGFRvIII-CAR-T cells that were able to migrate to the edges of GBM tumors, resulting in prolonged murine survival. The results were promising, suggesting that EGFRvIII-CAR-T cells can penetrate the blood-brain barrier and immune-privileged GBM environment.

Human Epithelial Receptor Type 2 (HER2) is another tyrosine kinase receptor implicated in gliomas, as HER2 expression is associated with de novo GBM and decreased survival.^33^ In 2010, Ahmed et al. aimed to evaluate the effectiveness of HER2-specific CAR-T cells created from patients with GBM.^34^ These CAR-T cells exhibited significant T cell proliferation and cytokine secretion, selectively killing HER2-positive GBM cells in vitro. Additionally, HER2-specific T-cell transfer led to significant tumor regression and improved survival in immunocompromised mice, but this response was relatively short-lived. Tumor recurrence was attributed to limited T cell persistence, which underscored the need to explore the impact of GSC immune evasion mechanisms on HER2-specific T cell activity. One such mechanism was subsequently studied, where third-generation HER2-CAR-T cells were used in combination with Programmed Cell Death Protein 1 (PD-1) inhibition to enhance the activation of CAR-T cells in vitro.^35^ Normally, PD-1 prevents the activation of cytotoxic T cells in tumors, which allows cancer cells to evade the immune system. However, when HER2-CAR-T cells and PD-1 blockade were combined, more interleukin-2 (IL-2) and interferon-gamma (IFN-γ) were released, which led to greater tumor elimination.

Another recently implicated GBM target is GD2, a disialoganglioside highly expressed in gliomas but in <4% of normal human nervous system tissue.^36,37^ Recently, GD2-CAR-T cells were developed that were effective in producing a specific antitumor response.^37^ When comparing intravenous (IV) and intracerebral (IC) delivery, IC injection of a single dose of GD2-CAR-T cells led to significantly improved survival compared to that of the control and IV groups. In another study, murine-derived CAR-T cells targeting GD2 demonstrated in vivo antitumor responses when the IV dose of GD2-CAR-T cells was followed by radiation therapy.^38^ Following these findings, another recent study by Gargett et al. found IV-administered GD2-CAR-T cells to be effective in an aggressive orthotopic xenograft model, especially when enhanced with an interleukin-15 transgene (meant to combat the immunosuppressive tumor environment).^39^ The authors speculated that the difference in IV efficacy between their results and those of Prapa et al. might have resulted from differences in manufacturing or in their CAR constructs. For example, Gargett et al. showed that different specialized nanomatrix and serum-free media were able to produce CAR-T cells with a more central memory phenotype that is theorized to improve CAR-T persistence.^40^ Regarding CAR construct differences, the intravenously effective GD2-CAR-T cells were third-generation constructs (with 2 costimulatory domains, Ox40 and CD28) while Prapa et al. developed second-generation constructs (with only one costimulatory domain, 4-1BB).^37,39^ Costimulatory domains like CD28 and 4-1BB may lead to a more rapid expansion of T cells and improved T-cell persistence,^10^ while Ox40 is an antigen-independent costimulatory domain that has been shown to improve 4-1BB-containing CAR-T cell proliferation and cytotoxicity, and decrease signs of exhaustion.^41^ Overall, the 2 studies established a promising role to further investigate GD2-targeting CAR-T cells in GBM.

### Clinical Studies

#### IL-13R*α*2

—There have been several completed clinical trials (Table 2) involving CAR-T therapy in GBM patients. In 2015, Brown et al. conducted the first CAR-T trial for GBM and targeted IL-13Rα2—a protein associated with poor prognosis and expressed in up to 50% of GBM cases—in 3 patients with recurrent disease.^42^ The patients, who consisted of 3 adults with recurrent or refractory grade III or IV gliomas that were amenable to resection, received 12 local infusions of IL-13Rα2-specific CD8 + T-cells following resection at the time of recurrence. Two patients had intracavitary infusions (8.6 and 10.3-month survival post-relapse), while one patient had both intracavitary and intraventricular treatment (13.9-month survival post-relapse). Encouragingly, no severe adverse events were observed, with magnetic resonance imaging suggestive of therapeutic responses. Two patients had recurrence in distant sites from where T-cell infusion occurred, and one patient had recurrence at an adjacent site, but with significantly decreased IL-13Rα2 expression here, suggesting a selective antitumor response. Overall, the study established the safety and potential efficacy of utilizing CARs targeting IL-13Rα2 in GBM. Future plans include improving T-cell persistence and determining the optimal delivery site for treatment.

**Table 2.** Completed CAR-T clinical trials in GBM.

Author/ year	*N*=	Type of T cell	Target	Delivery method	Adverse events (grade III or higher)	Median progression-free survival (months)	Median overall survival (months)
Brown et al. 2015	3	CTL	IL-13Rα2	IC, IVe	Headache, fatigue, neurological event	N/A	10.3 (post-relapse)
Brown et al. 2016	1	Memory	IL-13Rα2	IC, IVe	N/A	7.5	N/A
O’Rourke et al. 2017	10	Autologous	EGFRvIII	IV	Seizures, headache, weakness, hemorrhage	N/A	8
Ahmed et al. 2017	17	Virus-specific	HER2	IV	Confusion, cerebral edema	2.5	11.1
Keu et al. 2017	7	CTL	IL-13Rα2	Intratumoral, IVe	Headache, rash	N/A	7 (since first CTL infusion)
Goff et al. 2019	18	Autologous	EGFRvIII	IVe	Hypoxia, death	1.3	6.9
Vitanza et al. 2021	3	CTL	HER2	IC, IVe	Headache, pain, neurologic deficit, fever	N/A	N/A
Brown et al. 2022	6	Allogeneic CTL	IL-13Rα2	Intratumoral	N/A	N/A	2.9 (since first infusion)
Liu et al. 2023	8	Autologous	GD2	IV, IC	Seizure, headache	N/A	10 (post-infusion)

IC, intracavitary. IV, intravenous. IVe, intraventricular. CTL, cytotoxic lymphocyte.

A year later, the IL-13Rα2-CAR construct was updated to treat a 50-year-old male with recurrent and multifocal GBM.^43^ The patient received IL-13Rα2-CAR-T cells via intracavitary and intraventricular administration, for a total of 16 cycles. Although intracavitary administration prevented local recurrence, the development of new distant lesions led the researchers to attempt several cycles of intraventricular administration, which successfully led to significant regression of all intracranial and spinal tumors. The patient was also able to be weaned off systemic corticosteroids and returned to normal life. There were no toxic effects observed, although grade I or II events, including fever, headache, and fatigue, were reported within the first few days of treatment. The patient’s disease ultimately returned after 16 cycles (a progression-free survival of 228 days), at 4 new non-adjacent locations. Nonetheless, the clinical response was remarkable.

More recently, testing has been done for steroid-resistant, allogeneic “off-the-shelf” IL-13Rα2-targeting CAR-T cells.^44^ The rationale for developing allogeneic “off-the-shelf” products is decreased cost and increased adoptability compared to autologous CAR-T cells that require production for each individual patient.^44^ The allogeneic cells were developed by isolating peripheral blood mononuclear cells from a healthy male volunteer donor followed by transfer of an IL-13 zetakine plasmid and subsequent glucocorticoid receptor-targeting vector (to allow for concomitant glucocorticoid treatment, which is often necessary in GBM). Importantly, the cells did not show alloreactivity against a panel of various cell lines. Six patients with nonresectable, recurrent GBM were treated with intratumorally infused modified T-cells, IL-2, and dexamethasone over 2 weeks. Two patients had evidence of tumor regression at 4 weeks, while 2 others subsequently underwent craniotomies secondary to disease progression. The excised tumors showed evidence of treatment response solely in the area near the infusion site, as well as persistent expression of IL-13Rα2, which suggested failure of the treatment to eliminate the antigen. Median survival was 2.9 months after infusion. Fluorescence in situ hybridization analysis revealed that only a few cells remained at the 10-week post-infusion mark, indicating inadequate persistence. One patient showed a mild immune response to the CAR, possibly due to the low dose of dexamethasone. Of note, a first-generation CAR was used in this study. The authors hypothesized that modifications to the CAR to improve persistence, less culture and expansion, and targeting of the tumor immunosuppressive environment, could improve survival. Overall, the study provides a basis for further improving allogeneic, steroid-resistant IL-13Rα2-CAR-T cells.

#### EGFRvIII

There have been several attempts to target EGFRvIII in GBM using CAR-T cells. In 2017, O’Rourke et al. enrolled 10 patients with recurrent and multifocal MGMT-unmethylated (unmethylated status is known to confer a significantly lower overall survival^45^), EGFRvIII-expressing GBM.^46^ The patients in this study were treated with a single IV infusion of EGFRvIII-CAR-T cells. Patients who underwent surgical resection 2 weeks after infusion showed evidence of EGFRvIII-CAR-T cells in their tumors, whereas those who underwent surgical resection 3 months after infusion did not exhibit engraftment, which coincided with the peak detection of the CAR-T cells in the peripheral blood at 2 weeks. No adverse events related to off-target toxicity or CRS were observed. However, 3 patients experienced neurologic events, including seizures and neurological decline. It is worth noting that all patients had decreased EGFRvIII expression in their tumors, suggesting that the infusion did have its desired anti-target effect. While EGFRvIII-CAR-T cells were shown to traffic to the brain and proliferate, there was an increase in immunosuppressive molecules, likely representing tumor adaptation. Another barrier observed in this study was the natural heterogeneity of EGFRvIII expression.

In a subsequent phase I trial, which aimed to replicate results from other solid tumors by incorporating lymphodepletion and high doses of a third-generation EGFRvIII-CAR (>10^10^ cells), the median overall survival in 18 patients with recurrent GBM was 6.9 months, with no MRI-based tumor responses.^47^ At the highest T cell doses, 2 patients suffered severe dyspnea, with 1 developing severe hypotension and pulmonary edema and subsequently expiring. The presence of peripheral CAR-T cells at 1 month did not have a direct correlation with patient survival. Unfortunately, only one patient was progression-free at 6 months, and this patient had MGMT methylation, a marker associated with longer survival. These results mirrored the findings of the phase III Act IV trial, which found that an EGFRvIII vaccine was unable to improve survival.^48^ In this study, EGFRvIII elimination did not correlate with outcome. These findings raised the question of whether current EGFRvIII constructs or targeting EGFRvIII alone is sufficient to effectively treat GBM.

#### HER2

HER2 is another implicated target in gliomas. In a phase I clinical trial conducted in 2017, 17 patients with HER2-positive GBMs received HER2-CAR virus-specific T cells (VSTs) administered intravenously over a period of 12 weeks.^49^ Virus-specific T cells were employed to enhance T cell persistence via viral antigen engagement of native T-cell receptors. Although no maximum tolerated dose was reached, 2 patients experienced immune-related adverse events that were successfully managed supportively. While HER2-CAR VSTs were detected peripherally 1 year after infusion, there was no evidence of expansion. Nevertheless, 8 patients demonstrated benefits, including tumor regression or progression-free survival for some period of time. Median overall survival from the first infusion was 11.1 months. To improve outcomes, the researchers planned to explore the use of viral antigen vaccines and/or lymphodepletion in future studies.

In another trial, 3 young adults aged 19 to 26 who were diagnosed with glioma as children were treated with weekly infusions of HER2-CAR-T cells for 4 weeks.^50^ Two patients received intraventricular treatment and 1 received intracavitary treatment, without prior lymphodepletion. None of the patients experienced dose-limiting toxicity, but there were side effects including headache, pain at metastatic sites, and neurologic deficits. Despite the absence of detectable CAR-T cells post-infusion, magnetic resonance imaging showed evidence of inflammatory responses. However, this study did not report on progression-free survival or overall survival.

#### GD2

Most recently, in a clinical trial involving 8 patients with GD2-positive GBM, 5 patients demonstrated a partial response or stable disease following either IV or IV and intracavitary administration of GD2-CAR-T cells.^51^ Single and combined infusions were well tolerated, with 1 patient experiencing a grade II (moderate) seizure. Another finding of the study was an increased immunosuppressive environment with decreased GD2 expression in 1 patient who underwent resection after infusion.

Additionally, a recent phase I trial in diffuse midline gliomas, lethal pediatric central nervous system tumors, demonstrated the safety and efficacy of intravenous and intracerebroventricular GD2-CAR-T cells.^52^ Three of the four patients had clinical and radiographic improvement. Neurological symptoms relating to brainstem inflammation that the authors termed tumor inflammation-associated neurotoxicity were successfully treated via CSF diversion, osmotic agents, corticosteroids, and anti-cytokine agents such as anakinra. Furthermore, serum and CSF samples appeared to suggest successful inflammatory responses and increased cell-free tumor DNA following CAR-T treatment, indicating tumor lysis.

Active clinical trials, trials currently recruiting participants, and trials recruiting patients in the future are listed in Table 3.

**Table 3.** Ongoing and planned clinical trials of CAR-T cell therapies for GBM. Information obtained from Clinicaltrials.gov on October 24, 2023.

NCT #	Title	Phase	Sample characteristics	Target	Delivery method
NCT05868083	The Safety and Efficacy of SNC-109 CAR-T Cells Therapy the Recurrent Glioblastoma	1	Expecting 16 patients with recurrent GBM		IV
NCT05627323	CAR T Cells in Patients With MMP2 + Recurrent or Progressive Glioblastoma	1	42 estimated patients with recurrent/refractory GBM	Chlorotoxin (CLTX)	IC, IVe
NCT05577091	Tris-CAR-T Cell Therapy for Recurrent Glioblastoma	1	10 patients with recurrent GBM, not yet recruiting	CD44, CD133	IC
NCT05353530	Phase I Study of IL-8 Receptor-modified CD70 CAR T Cell Therapy in CD70 + and MGMT-unmethylated Adult Glioblastoma (IMPACT)	1	18 estimated patients with newly diagnosed GBM	CD70	IV
NCT04385173	Pilot Study of B7-H3 CAR-T in Treating Patients With Recurrent and Refractory Glioblastoma	1	12 estimated patients with recurrent/refractory GBM	B7-H3	IC, IVe
NCT05241392	Safety and Efficacy Study of Anti-B7-H3 CAR-T Cell Therapy for Recurrent Glioblastoma	1	30 estimated patients with recurrent GBM	B7-H3	IC, IVe
NCT04003649	IL13Ra2-CAR T Cells With or Without Nivolumab and Ipilimumab in Treating Patients With GBM	1	60 estimated patients with recurrent/refractory GBM	IL-13Rα2	IC, IVe
NCT04214392	Chimeric Antigen Receptor (CAR) T Cells With a Chlorotoxin Tumor-Targeting Domain for the Treatment of MMP2 + Recurrent or Progressive Glioblastoma	1	36 estimated patients with recurrent/refractory GBM	CLTX	IC, IVe
NCT05366179	Autologous CAR-T Cells Targeting B7-H3 in Recurrent or Refractory GBM CAR.B7-H3Tc	1	36 estimated patients with recurrent/refractory GBM	B7-H3	IVe
NCT05835687	Loc3CAR: Locoregional Delivery of B7-H3-CAR T Cells for Pediatric Patients With Primary CNS Tumors	1	36 estimated pediatric patients with primary CNS neoplasms, including GBM	B7-H3	IC
NCT04661384	Brain Tumor-Specific Immune Cells (IL13Ralpha2-CAR T Cells) for the Treatment of Leptomeningeal Glioblastoma, Ependymoma, or Medulloblastoma	1	30 estimated patients with leptomeningeal disease from GBM, ependymoma, medulloblastoma	IL-13Rα2	IC, IVe
NCT05802693	A Study to Evaluate the Safety, Tolerance and Initial Efficacy of EGFRvIII CAR-T on Glioblastoma	1	22 estimated patients with recurrent GBM, not yet recruiting	EGFRvIII	IC
NCT02208362	Genetically Modified T-cells in Treating Patients With Recurrent or Refractory Malignant Glioma	1	82 patients enrolled with recurrent/refractory gliomas	IL-13Rα2	IC, IVe
NCT05660369	CARv3-TEAM-E T Cells in Glioblastoma	1	21 estimated patients with newly diagnosed/ recurrent GBM	EGFRvIII	IVe

## Challenges and Future Directions

### Antigen Escape

Several challenges exist in implementing CAR-T therapy for GBM. The first challenge is tumor heterogeneity, defined as the expression of different antigens among the subpopulations of a tumor, which contributes to antigen escape.^42,46,53–55^ Antigen escape refers to tumors losing expression of a targeted antigen (eg, loss of IL-13Rα2 following IL-13Rα2-CAR-T therapy or EGFRvIII loss following EGFRvIII-CAR-T therapy).^43,46^ By employing single-cell RNA sequencing (RNA-seq) to profile cells from 5 GBM samples, Patel et al. demonstrated GBM intratumoral heterogeneity based on transcriptional programming and degree of stemness. The authors also noted implications for prognosis.^54^ Meanwhile, DNA barcoding has demonstrated the importance of GSCs in hierarchically establishing tumoral heterogeneity.^55^ This provides further motivation for targeting the GSC population. An Immunohistochemistry study demonstrated that in high-grade (WHO grade III–IV) glioma samples, there was a clustering of antigen expression into “distinct neighborhoods.”^53^ For example, in areas of pseudopalisading necrosis, IL-13Rα2 and HER2 were expressed, but EGFR was not. This suggests a role for IL-13Rα2 and HER2 in hypoxic conditions. Furthermore, there appeared to be a reciprocal relationship between the expression of EGFR and the combination of IL-13Rα2 and HER2. Other preclinical data have suggested that IL-13Rα2 and EGFRvIII may interact to promote GBM proliferation.^56^ Thus, CARs targeting single antigens may be insufficient due to antigen escape.

Strategies to address antigen escape include targeting multiple antigens simultaneously, finding novel and more ubiquitously expressed antigens, and targeting GSCs. One study first mathematically postulated that simultaneously targeting HER2 and IL-13Rα2 would improve the antitumor effects of CAR-T therapy.^57^ The authors then designed a tandem CAR-T (TanCAR) that targeted both HER2 and IL-13Rα2 simultaneously.^58^ They compared survival in groups receiving TanCAR, combinations of CARs targeting single antigens (CARpool), and T cells expressing both CARs but not in tandem (biCAR). TanCAR featured significantly improved immune activation and survival in vivo (100% of animals survived at 140 days after treatment compared to 0% and 30% in the CARpool and biCAR groups, respectively). A different tandem CAR-T cell targeted EGFRvIII and IL-13Rα2 and showed improved antitumor effects when targeting both antigens simultaneously in heterogeneous GBM.^59^ One group expanded on this concept by designing a trivalent CAR-T cell to target 3 antigens: HER2, IL-13Rα2, and ephrin-A2 (EphA2).^60^ The authors used flow cytometry for 15 primary GBM samples to highlight the inter-patient heterogeneity for these antigens. They then demonstrated that the trivalent CAR-T cells (named UCAR-T cells) lysed cells expressing any 1 of the 3 antigens. In orthotopic murine models, intratumorally injected UCAR-T cells led to significantly improved survival compared to bispecific and single CAR-T cells.^60^

Finding better, more ubiquitously expressed antigens may also prevent antigen escape. For example, exploiting gliomas’ affinity for chlorotoxin (CLTX) led to the development of a chlorotoxin-targeting CAR-T cell (CLTX-CAR-T).^61^ Chlorotoxin binding was observed in almost all 23 patient samples and was independent of IL-13Rα2, HER2, and EGFR expression. In vivo, intraventricular and intracranial delivery of CLTX-CARs led to tumor regression and improved survival, while intravenous delivery was ineffective. Furthermore, off-target effects were minimal. Several ongoing CAR-T clinical trials involving chlorotoxin are highlighted in Table 3. In the future, it would be interesting to compare CLTX-CAR-T to other CARs, including bivalent and trivalent constructs. A similar recent study used an antibody library to discover new, specific GBM targets.^62^ There were 2 clones that bound to GBM and avoided native brain tissue. One of the clones was discovered to bind to B7-H3, a molecule implicated in immunosuppression.^62,63^ Promisingly, a B7-H3 targeting CAR was able to lyse GBM cells.^62^ This has culminated in several ongoing studies involving B7-H3-CAR-T cells in GBM (Table 3).^64^

CD70 is another promising antigen that is expressed more frequently in recurrent GBM, is specific to tumoral tissue, and is also associated with reduced survival.^65^ CD70 is a transmembrane glycoprotein and a member of the tumor necrosis factor superfamily.^66^ This antigen correlates with mesenchymal behavior and is hypothesized to promote GBM microenvironment immunosuppression via M2 macrophage recruitment and CD8 + T cell death.^65–67^ Thus, Jin et al. hypothesized that targeting CD70 might be therapeutic, which an in vivo CAR construct confirmed.^65^ A subsequent study then also demonstrated CD70-CAR-T efficacy in vitro and in vivo with improved survival.^66^ The authors also noted T cell exhaustion from continuous CD70/CD27 interaction, which led them to speculate that combining CD70-CAR-T cells with other immunostimulatory therapies might be highly effective. For example, tumors appear to overexpress the immunosuppressive chemokine IL-8, and ionizing radiation can also upregulate IL-8.^11^ Jin et al. used these properties to create a CD70-targeting CAR with a modified IL-8 receptor (CXCR1 or CXCR2), called 8R-70CAR. An interesting finding was that the duration of intratumoral persistence of the 8R-70CAR T cells was more predictive of survival, as opposed to the duration of peripheral persistence. The 8R-70CAR-T cells demonstrated enhanced migratory and proliferative capacities resulting in complete tumor regression of advanced-stage tumors, such as GBM, ovarian, and pancreatic cancers, compared to the nonmodified CD70-CAR-T cells.^11^ This research is culminating in a phase I clinical trial involving 8R-70CAR T cells for newly diagnosed GBM (NCT05353530), which is recruiting now at University of Florida.^68^

### Immunosuppressive Tumor Environments

Other implicated immunosuppressive cytokines include IL-15 receptor alpha (IL-15Rα) and CXCL11. IL-15Rα was found to be expressed on myeloid-derived suppressor cells.^69^ Adding IL-15 to a IL-13Rα2 CAR construct essentially reversed the immunosuppressive environment in GBM and reduced MDSC and glioma cell populations, which led to improved murine survival. Similarly, another study leveraged the chemokine CXCL11 to improve B7H3-CAR-T infiltration.^70^ The authors created an adenovirus expressing CXCL11 that helped improve the infiltration of B7H3-CAR-T cells in an immunocompetent murine model, which led to greater antitumor effects. The tumor immunosuppressive environment was reprogrammed because of the adenovirus and contained greater levels of natural killer cells and M1-like macrophages.

The immune checkpoint PD-1/PD-L1 axis may also be implicated in GBM-related immunosuppression.^71^ CRISPR/Cas9 gene editing to target PD-1 in EGFRvIII-CAR-T cells improved cancer growth inhibition in vitro and in vivo.^72,73^ Intracranial injection of EGFRvIII-CAR-T cells with deleted PD-1 led to significantly improved survival, which further suggests a role for the PD-1/PD-L1 axis. However, Tang et al. used patient samples from O’Rourke’s phase I clinical study^46^ and demonstrated a positive association between PD-1 expression and peripheral engraftment of EGFRvIII-CAR-T cells, as well as improved progression-free survival.^74^ Thus, more studies are needed to clarify the effects of PD-1 and its potential role in mediating CAR-T effectiveness in GBM.

### Vascular Dysfunction

Some efforts have focused on the importance of peritumoral vasculature to improve CAR-T therapy penetration in GBM.^75,76^ p21-activated kinase 4 has been identified as a contributor to endothelial cell dysfunction, specifically in the GBM tumor vascular environment.^75^ p21-activated kinase 4 inhibition improved T cell infiltration and increased survival in murine models when combined with EGFRvIII-CAR-T therapy. Meanwhile, a recent study implicated phosphoglycerate dehydrogenase (PHGDH) metabolism in endothelial cells as a driver of vascular dysfunction in GBM.^76^ PHGDH inhibition sensitized murine GBM models to EGFRvIII-CAR-T therapy, which led to improved survival and T-cell persistence. Most recently, vascular endothelial growth factor inhibition improved the delivery of EGFRvIII-CAR-T cells.^77^ Thus, combining tumoral vasculature antagonists with CARs may be worthwhile in future trials.

### Access

Another consideration for future implementation of CAR-T therapy is cost and availability. One strategy involves making generic, readily available products.^44,73^ CRISPR/Cas9—which has the ability to target multiple genes simultaneously—was used to create a generic EGFRvIII-CAR-T cell that prevents rejection by disrupting the endogenous T-cell receptor and beta-2 microglobulin.^73^ Similarly, Brown et al. used zinc finger nuclease targeting to make a generic, steroid-resistant IL-13Rα2-CAR product.^44^ The intracranially administered CAR-T cells showed responses in 4 of the 6 patients, with good tolerability. Further modifications will hopefully lead to improved efficacy of these products, with more widespread accessibility compared to autologous products.

## Conclusion

This paper has summarized landmark clinical trials, emerging science, and future directions involving CAR-T therapy in the treatment of GBM. More research, likely combining several of the above strategies—including multi-antigen CAR-T cells and checkpoint immunotherapy—will hopefully lead to continued improvements of this revolutionary therapy and marked improvements in the survival of patients with GBM.

## Data Availability

The data generated in this study will be made available by reasonable request by contacting the corresponding author.
